# The Eurasian otter (*Lutra lutra*) as a potential host for rickettsial pathogens in southern Italy

**DOI:** 10.1371/journal.pone.0173556

**Published:** 2017-03-07

**Authors:** Mario Santoro, Nicola D’Alessio, Anna Cerrone, Maria Gabriella Lucibelli, Giorgia Borriello, Gaetano Aloise, Clementina Auriemma, Nunzia Riccone, Giorgio Galiero

**Affiliations:** 1 Istituto Zooprofilattico Sperimentale del Mezzogiorno, Portici (Naples), Italy; 2 Museo di Storia Naturale della Calabria e Orto Botanico, Università della Calabria, Rende (Cosenza), Italy; Johns Hopkins University, UNITED STATES

## Abstract

Canine monocytic ehrlichiosis and rickettsiosis are zoonotic tick-borne diseases of canids caused by the intracellular obligate bacteria *Ehrlichia canis* and *Rickettsia* species respectively. In this study, we investigated using standard and real-time PCR and sequencing, the occurrence and molecular characterization of *E*. *canis* and *Rickettsia* species in the Eurasian otter (*Lutra lutra*) from the southern Italian population. Samples were screened by using molecular assays also for *Neospora caninum*, *Toxoplasma gondii*, *Clamydophyla* spp., *Coxiella burnetii*, *Leishmania* spp., *Cryptosporidium* spp., and *Giardia* spp. detection, and helminths were studied by traditional methods. Out of six carcasses tested, three were positive for *E*. *canis* and co-infection with *Rickettsia* sp. occurred in one of those. Sequences of the 16S rRNA *E*. *canis* gene were identical to each other but differed from most of those previously found in red foxes (*Vulpes vulpes*) and wolves (*Canis lupus*) from southern Italy. Helminths included just cystacanths of *Sphaerirostris* spp. from the intestine of two Eurasian otters and the nematode *Angiostrongylus vasorum* from the lungs of a single Eurasian otter. None of the samples was positive for the other investigated selected pathogens. This study is the first report on the evidence of infection by rickettsial pathogens in the Eurasian otter. The present result prompts some inquiries into the pathogenic role of those bacteria for the isolated sub-populations of the endangered Eurasian otter in southern Italy.

## Introduction

Canine monocytic ehrlichiosis (CME) and rickettsiosis are zoonotic tick-borne diseases of canids caused by the intracellular obligate bacteria *Ehrlichia canis* and *Rickettsia* species respectively [1–5]. Both CME and many *Rickettsia* spp. are endemic in all European countries bordering the Mediterranean Sea [3,4,6]. Among rickettsial pathogens of the spotted fever group, *R*. *conorii* is the most common being the causative agent of the Mediterranean spotted fever in humans [2]. The brown dog tick *Rhipicephalus sanguineus* sensu lato (s.l.) is generally accepted as the main vector for both *E*. *canis* and *R*. *conorii* infections and suspected as the main reservoir for *R*. *conorii* [2,3].

Molecular surveys in European wildlife reported *E*. *canis* detection only from red foxes (*Vulpes vulpes*) [7–10], although recently this bacterium has been detected in red foxes and wolves (*Canis lupus*) from southern Italy [5]. The high prevalence of infection found in wildlife carnivores in this latter study suggests that a sylvatic life cycle of this pathogen may occur. Other species of reservoir hosts remain to be identified, as the southern regions of Italy show an incidence risk of infection for CME higher than other areas in southern Europe [6]. Similarly, it was suggested that rodents and other small mammals may act as reservoir hosts for *Rickettsia* species [11] and several tick species may harbor a wide range of *Rickettsia* spp. [12,13].

The Italian population of the Eurasian otter (*Lutra lutra*) (about 250 adult individuals) occurs in the southern part of the peninsula with two isolated sub-populations including a larger one occurring in Campania, Basilicata, Calabria and Puglia regions, and a smaller one in Molise and Abruzzo regions [14]. To our knowledge no data exists on arthropod-borne pathogens of Eurasian otters except for the occasional finding of the heart nematode *Dirofilaria immitis* [15]. It was demonstrated by PCR assay that a related host species, the American river otter (*Lontra canadensis*) may be infected with *Babesia* spp., *Ehrlichia ewingii* and *Bartonella* spp. [16]. Chinnadurai et al. [16] suggested that despite the rarely detection of ticks infesting the American river otter the potential risk for vector-associated pathogens may be greater than previously appreciated. Recently *Bartonella* spp. associated with unusually high mortality event were isolated from the northern and southern sea otters (*Enhydra lutris kenyoni* and *Enhydra lutris nereis*) in Alaska [17].

Here, we perform a wide survey of selected pathogens by traditional and molecular tools on six carcasses of the Eurasian otter from the southern Italian population and demonstrate molecularly that they can be naturally infected by *E*. *canis* and *Rickettsia* sp.

## Materials and methods

### Otter samples and diagnostic procedures

The Istituto Zooprofilattico Sperimentale del Mezzogiorno is accredited by the Italian Ministry of Health to perform systematic surveys on infectious diseases of wildlife. Procedures for this study were performed in accordance with the guide for the care and use of wildlife by the Italian Ministry of Health.

Six carcasses of road-killed Eurasian otters from three regions of southern Italy obtained between November 2014 and December 2016 were examined for selected pathogens (Tables 1 and 2). Carcasses were sexed and on the basis of morphological features and tooth examination, were classified as juveniles (<12 months old) or adults (>12 months old) [18]. Following the post-mortem examination, the trachea, bronchi, lungs, heart, great vessels, urinary bladder, liver, gall bladder, kidneys, oesophagus, stomach, and intestine of each Eurasian otter were examined separately for helminths. Organs were sectioned and surfaces examined visually, then washed through a series of 50 and 100-mesh screens. The remaining washed material from each organ was then examined carefully under a dissecting microscope and helminths were collected, counted and preserved in 70% ethanol. Acanthocephalans and nematodes were cleared in lactophenol on a glass slide for identification and then returned to the preservative. Standard sedimentation and flotation methods were used to detect parasite eggs and oocysts from feces. The tongue and diaphragm from each Eurasian otter were examined for *Trichinella* spp. by artificial pepsin digestion [19].

**Table 1:** Signalament (sex: F, female; M, male; weight in kilograms and age: J, juvenile; A, adult) and geographical origin of six Eurasian otters (*Lutra lutra*) obtained from southern Italy.

Individual ID	Sex	Weight	Age	Site of collection	Date of collection
IZSM79797	F	5 kg	J	Capaccio, Salerno province, Campania region	April 2016
IZSM79829	M	4.8 kg	J	Capaccio, Salerno province, Campania region	March 2016
IZSM79844	M	5.2 kg	J	407 Basentana state road close to the Balzano bridge junction, Matera province, Basilicata region	November 2014
IZSM89305	M	5.8 kg	A	Crati River in Thurio (Corigliano Calabro), Cosenza province, Calabria region	March 2014
IZSM130699	F	4.2 kg	A	Capaccio, Salerno province, Campania region	April 2016
IZSM160224	M	5.6 kg	A	283 state road, San Marco Argentano, Cosenza province, Calabria region	December 2016

**Table 2:** Primers used in the present study and related information.

Target pathogen	Target tissue	Gene ID	Primer ID	Primer sequence (5’-3’)	References
*Ehrlichia canis*	Spleen	16S rRNA	Forward (Elri)	TCGCTATTAGATGAGCCTACGT	[20]
			Reverse (Elri)	GAGTCTGGACCGTATCTCAG	
*Rickettsia* spp.	Spleen	17-kD antigen	Tz-15-19	TTCTCAATTCGGTAAGGGC	[24]
			Tz-16-20	ATATTGACCAGTGCTATTTC	
*Coxiella burnetii*	Spleen	IS111	Forward (RT Coxi)	AAAACGGATAAAAAGAGTCTGTGGTT	[34,35]
			Reverse (RT Coxi)	CCACACAAGCGCGATTCAT	
			Probe (RT Coxi)	FAM-AAAGCACTCATTGAGCGCCGCG-TAMRA	
*Neospora caninum* and *Toxoplasma gondi*	Heart, brain	16 S rRNA	APIF	AAGTATAAGCTTTTATACGGC	[36]
			APIR	CACTGCCACGGTAGTCCAATAC	
*Giardia* spp.	Feces	16S rRNA	Forward (GD80)	GACGGCTCAGGACAACGGTT	[37,38]
			Reverse (GD127)	TTGCCAGCGGTGTCCG	
			Giardia Probe	TET-CCCGCGGCGGTCCCTGCT-TAMRA	
*Cryptosporidium* spp.	Feces	Cowp	Forward (COWP)	CAAATTGATACCGTTTGTCCTTCTG	[39,40]
			Reverse (COWP)	GGCATGTCGATTCTAATTCAGCT	
			Crypto Probe	FAM-TGCCATACATTGTTGTCC-TAMRA	
*Leishmania* spp.	Spleen	Minicircle DNA footprint -	QLK2-U	GGCGTTCTGCGAAAACCG	[39,40]
		kinetoplast	QLK2-D	AAAATGGCATTTTCGGGCC	
			Q-Leish Probe	FAM-TGGGTGCAGAAATCCCGTTCA	
*Chlamydophyla* spp.	Lungs	23S rRNA	Forward Ch23S	CTG AAACCAGTAGCTTATAAGCGGT	[41–43]
			Reverse Ch23S	ACC TCGCCGTTTAACTTAACT CC	
			Ch23S Probe	FAM-CTCATCATGCAAAAGGCACGCCG-TAMRA	

### Molecular analyses

For each carcass, a sample of feces and several tissues including brain, spleen, lung, and heart was stored at -20°C and examined selectively for molecular analyses as reported in Table 2. Genomic DNA was extracted from samples using a commercial kit (QIAamp DNA mini kit, Qiagen, Hilden, Germany) according to the manufacturer’s protocol.

*E*. *canis* detection was performed from spleen samples by a real-time PCR analysis (rt PCR) targeting the 16S rRNA gene (124 bp) as described by Waner et al. [20] with slight modifications [5]. Briefly, reaction was performed in a total volume of 20 μL containing SsoFast^TM^ EvaGreen^®^ Supermix 1X (Biorad Laboratories, Hercules, CA, USA), 0.6 μM of each primer and 2 μL of DNA. The primers used were 16S-F (5′-TCGCTATTAGATGAGCCTACGT-3′) and 16S-R (5′-GAGTCTGGACCGTATCTCAG-3′). Amplification conditions included an initial denaturation for 30 sec at 95°C, followed by 40 cycles of denaturation at 95°C for 10 sec, annealing and extension at 60°C for 10 sec. Amplicons were subsequently subjected to a melt step by raising the temperature to 95°C at a rate of 0.5°C each 5 sec. Amplification and melt profiles were carried out and analyzed using the CFX96 system (BioRad Laboratories, Hercules, CA, USA). A positive control containing genomic DNA for *E*. *canis*, and a negative and non-template control (NTC) were included in each PCR reaction. Plasmid DNA including a 480bp fragment of the 16S rRNA gene from *E*. *cani*s C2 was provided by the Italian Reference Centre for *Anaplasma*, *Babesia*, *Rickettsia*, and *Theileria* (C.R.A.Ba.R.T.). In order to obtain longer DNA fragments (389 bp), the *E*. *canis*-positive samples were amplified by a nested-PCR protocol, as previously described [21] with some modifications. Briefly, in the first amplification round, the final reaction volume was 50 μl containing Hot Star Taq Master Mix 1X (Qiagen, Hilden, Germany), a final concentration of 2 mM Mg^2+^, 0.2 μM of each primer (forward ECC 5′-AGAACGAACGCTGGCGGCAAGCC-3′ and reverse ECB 5′-CGTATTACCGCGGCTGCTGGCA-3′) and 3 μl of DNA. The thermal profile consisted of an initial activation step at 95°C for 15 min, followed by 40 cycles of 94°C for 1 min, 60°C for 1 min, and 72°C for 1 min. Nested PCR was performed with primers HE3 (5′-TATAGGTACCGTCATTATCTTCCCTAT-3′) and “canis” (5′-CAATTATTTATAGCCTCTGGCTATAGGA-3′) by using 3 μl of the product from the outside amplification. The second thermal profile was the same as used for the first amplification. PCR products were resolved by the QiaXcel automated capillary electrophoresis system according to the manufacturer’s recommendations (QIAGEN, Hilden, Germany). Amplicons were purified and sequenced in both directions using the same primers as for PCR, employing the Big Dye Terminator Cycle Sequencing Kit v1.1 (Thermo Fischer Scientific, USA) in an automated sequencer (ABI-PRISM 377). Sequences were aligned using ClustalW program [22] and compared with those available in GenBank (BLAST– http://blast.ncbi.nlm.nih.gov/Blast.cgi). The percentage of nucleotide variation among sequences was calculated by the pairwise sequence alignment using the EMBOSS Stretcher program (http://www.ebi.ac.uk/Tools/psa/emboss_stretcher/) [23].

Tissue samples were also screened by classical PCR assays to amplify specific genes of *Neospora caninum*, *Toxoplasma gondii*, and *Rickettsia* spp., and by rt PCR for *Chlamydophyla* spp., *Coxiella burnetii*, *Cryptosporidium* spp., *Giardia* spp., and *Leishmania* spp. detection. Primers and target tissues used in this study are listed in Table 2, and specific amplification reaction conditions are those reported in the specific references.

## Results

Three (ID 79797; 79829; 130699) out of six carcasses were positive to *E*. *canis* by rt PCR, all coming from Capaccio (Salerno province), and one of those (ID 79829) was also positive for *Rickettsia* sp. by standard PCR (Table 1). *E*. *canis* and the *Rickettsia* positive samples were amplified by nested PCR for further sequencing analysis. Among the three *E*. *canis*-positive individuals, we successfully sequenced DNA from two individuals (ID 79797; 79829). Sequenced amplicons obtained were deposited in GenBank under accession numbers KY559099 and KY559100 for *E*. *canis* and KY559098 for *Rickettsia* sp.

*E*. *canis* sequenced amplicons were identical and they showed a high nucleotide similarity (99.4%) with *E*. *canis* 16S rRNA gene sequences found in a dog (GQ857078) and foxes and wolves (KX371787, 99.5%; KX371788, 99%; KX371789, 99%) and 100% similarity with an *E*. *canis* 16S rRNA gene sequence found in red foxes from southern Italy (KX371786) available in GenBank. Single nucleotide polimorphisms among genotypes of *E*. *canis* found in wildlife mammals from southern Italy compared to a reference strain from a dog from Sicily are listed in Table 3. *Rickettsia* sequenced amplicon exhibited 99% similarity with both *R*. *rickettsii* (CP000766.3) and *R*. *conorii* (JN182793.1) available in GenBank.

**Table 3:** Single nucleotide polimorphisms among genotypes of *Ehrlichia canis* found in wildlife mammals from southern Italy compared to a reference strain from a dog.

GenBank accession number	Nucleotide positions*					Host	References
	185	235	252	383	452		
GQ857078	C	A	C	T	C	Dog	[44]
KX371786	-	A	C	T	T	Red fox, wolf	[5]
KX371787	-	T	C	T	T	Red fox	[5]
KX371788	-	T	T	T	T	Red fox	[5]
KX371789	-	T	C	C	T	Red fox	[5]
KY559099, KY559100	A	A	C	-	-	Eurasian otter	[This study]

* -: nucleotide position not included in the sequenced amplicon

The samples were negative to the molecular detection of the other pathogens included in this survey. Helminths included two specimens of the nematode *Angiostrongylus vasorum* from lungs of a single Eurasian otter (ID 160224) and cystacanths of the acanthocephalan *Sphaerirostris* spp. in two Eurasian otters with 9 (ID 79844) and 117 (ID 79829) individual parasites respectively. Coprological and *Trichinella* examinations were negative.

## Discussion

This is the first study reporting evidence of an infection by rickettsial pathogens in the Eurasian otter. Although based on a limited number of carcasses, we demonstrated by molecular methods that the Eurasian otter can be naturally infected by *E*. *canis* and *Rickettsia* sp., however if the Eurasian otter plays a role in the maintenance of their sylvatic cycles or represents an accidental host remains unknown.

The sequences of *E*. *canis* here found were identical to each other but differed from most of those previously found in foxes and wolves from southern Italy [5], confirming high intra-species variability of *E*. *canis*, irrespective of the geographical origin. The molecular assay here used for *Rickettsia* detection is specific for *R*. *conorii* and *R*. *rickettsii* but is unable to differentiate the two species since these are highly related [24]. In accordance with Telford and Goethert [4], *R*. *conorii* is the causative agent of Mediterranean spotted fever in the Mediterranean region, and east, central and southern Africa, in contrast *R*. *rickettsii* causes Rocky Mountain Fever in North America. Among these two *Rickettsia* species *R*. *conorii* is the only one found molecularly in the Old World [4].

The present result prompts some inquiries into the pathogenic role of those bacteria in this new host. Because in dogs, CME and rickettsiosis may cause severe disease characterized by profound hematological changes, acute febrile illness, anorexia, and emaciation until death [3,25], the main concern is those infections may be important in the survival of the Italian population of the Eurasian otter. The only clinical data of natural infection by *E*. *canis* in a wildlife species was by Harvey et al. [26] describing an epizootic in captive wolves, dogs, and wolf-dog crosses from a small zoo in north central Florida associated with a massive *R*. *sanguineus* s.l. infestation. Five of nine adult canids and all eight pups confined to a common kennel died as a result of the infection. Specifically, two adult wolves infected showed depression, anorexia, weight loss, fever and epistaxis until a fatal cachexia [26]. With regard to the helminth parasites, in southern Italy *A*. *vasorum* is a common pathogenic pulmonary nematode of red fox [27]; definitive hosts of *Sphaerirostris* spp. are birds while cystacanths are found in tissues of amphibians and reptiles [28,29]. The presence of immature forms of *Sphaerirostris* spp. in the intestine of the Eurasian otter suggests that this mammal species is not a suitable host for the parasite.

*R*. *sanguineus* sensu lato (s.l.), the primary vector of CME and *R*. *conorii*, in areas where it occurs rarely parasitizes hosts other than dogs [30]. Besides *R*. *sanguineus* s.l. and *Dermacentor variabilis* [3], *Dermacentor marginatus* and *Ixodes canisuga* have also been recently suggested as vectors of *E*. *canis* [31], and *R*. *conorii* was recently detected in *Rhipicephalus turanicus* and *Ixodes ricinus* from a city park in Rome (Italy) [13].

Studies on ticks of Eurasian otters reported just three species within the genus *Ixodes* (*I*. *canisuga*, *I*. *hexagonus*, and *I*. *ricinus*) with low prevalence and intensity of infestation [32,33]. *I*. *hexagonus* is the only species infesting the Eurasian otter from England and Wales (199 out of 820 otters with mean intensity of 7.2) [33]. Of the three species found in Eurasian otters from Germany the most prevalent was *I*. *hexagonus* (27 out of 541 otters with mean intensity of 8) following by *I*. *canisuga* and *I*. *ricinus* both found in a single individual otter with three and six tick specimens respectively [32]. Unfortunately, we did not find ticks from carcasses and no data is available on ticks of Eurasian otters from Italy. Further studies on both tick species as well as occurrence and molecular detection of arthropod-borne pathogens in hosts (including trapped hosts for blood collection) and their ectoparasites are needed to predict entomological risk for the Eurasian otter and in general for wildlife and humans and to determine which vector species may be involved in the sylvatic cycle of *E*. *canis* and *Rickettsia* sp. in southern Italy.
